# MicroRNA-mediated translational pathways are regulated in the orbitofrontal cortex and peripheral blood samples during acute abstinence from heroin self-administration

**DOI:** 10.3389/adar.2023.11668

**Published:** 2023-08-14

**Authors:** Mary Tresa Zanda, Leila Saikali, Paige Morris, Stephanie E. Daws

**Affiliations:** ^1^ Center for Substance Abuse Research, Temple University, Philadelphia, PA, United States; ^2^ Department of Neural Sciences, Temple University, Philadelphia, PA, United States; ^3^ College of Liberal Arts, Temple University, Philadelphia, PA, United States

**Keywords:** microRNA, heroin, opioid, biomarker, self-administration

## Abstract

Opioid misuse in the United States contributes to >70% of annual overdose deaths. To develop additional therapeutics that may prevent opioid misuse, further studies on the neurobiological consequences of opioid exposure are needed. Here we sought to characterize molecular neuroadaptations involving microRNA (miRNA) pathways in the brain and blood of adult male rats that self-administered the opioid heroin. miRNAs are ∼18–24 nucleotide RNAs that regulate protein expression by preventing mRNA translation into proteins. Manipulation of miRNAs and their downstream pathways can critically regulate drug seeking behavior. We performed small-RNA sequencing of miRNAs and proteomics profiling on tissue from the orbitofrontal cortex (OFC), a brain region associated with heroin seeking, following 2 days of forced abstinence from self-administration of 0.03 mg/kg/infusion heroin or sucrose. Heroin self-administration resulted in a robust shift of the OFC miRNA profile, regulating 77 miRNAs, while sucrose self-administration only regulated 9 miRNAs that did not overlap with the heroin-induced profile. Conversely, proteomics revealed dual regulation of seven proteins by both heroin and sucrose in the OFC. Pathway analysis determined that heroin-associated miRNA pathways are predicted to target genes associated with the term “prion disease,” a term that was also enriched in the heroin-induced protein expression dataset. Lastly, we confirmed that a subset of heroin-induced miRNA expression changes in the OFC are regulated in peripheral serum and correlate with heroin infusions. These findings demonstrate that peripheral blood samples may have biomarker utility for assessment of drug-induced miRNA pathway alterations that occur in the brain following chronic drug exposure.

## Introduction

Misuse of opioid drugs is associated with a high risk of overdose death [1]. A drastic increase in the incidence of misuse and overdose of opioids has occurred in the United States over the past two decades [2] and represents a major public health concern. In 2021, opioids were involved in more than 70% of the 100,000+ overdose deaths that occurred in the United States [3]. These epidemiological patterns emphasize that critical efforts are required to reduce drug overdose deaths and aid in maintenance of abstinence behavior from opioid use. Because many patients recovering from opioid use disorder (OUD) continue to experience motivation to seek opioids, despite abstinence or FDA-approved OUD medications [4, 5], elucidation of the molecular signaling patterns in the drug-free period following cessation of drug use may provide insight into the pathways that can be targeted for reduction of drug seeking behavior.

Rodent models of drug self-administration provide an excellent tool to model drug seeking behavior and interrogate the molecular neuroadaptations that arise in discrete brain areas following chronic drug exposure. Previous studies from our labs and others have demonstrated that chronic self-administration of opioids, such as morphine or heroin, induces drug seeking behavior that is both immediate and long lasting in the absence of drug access [6–9]. Such behaviors are accompanied by regulation of a class of small noncoding RNAs called microRNAs (miRNAs) that are ∼18–24 nucleotides long [10]. miRNAs can regulate gene expression by inhibiting translation of a “target” mRNA to protein [10]. The short sequence of miRNAs allows them to accomplish this process by binding to the 3′-UTR of a target mRNA with sequence complementarity and inducing deadenylation of poly-A mRNA [10]. miRNAs bind to their targets within a short 6–8 nucleotide “seed” region, which theoretically permits an individual miRNA to target 100s, even 1000s of mRNA sequences [11]. Because of this, miRNA-mediated inhibition of protein translation is an essential regulatory mechanism for modulation of gene expression and the proteome [11]. Exposure to all classes of drugs can induce long-lasting alterations to brain miRNA expression profiles and regulation of miRNA function can modulate drug seeking behavior [7, 12–24]. Manipulation of individual miRNA expression or functional capabilities has been reported to regulate seeking for the opioids morphine and heroin, as well as psychostimulants and alcohol [14, 17, 20, 21, 23, 24]. In our recent publication, we reported the regulation of miRNAs and their associated downstream proteins in the orbitofrontal cortex (OFC) of rats following late abstinence (21 days) from self-administration of two heroin dose, 0.03 mg/kg/infusion or 0.075 mg/kg/infusion [24]. OFC-specific manipulation of the heroin-associated miRNA miR-485-5p resulted in regulation of long-lasting heroin seeking behavior [24]. The OFC has been identified as a key brain region that is active during incubation of heroin craving [25] and humans that have used heroin chronically display elevated blood flow to the OFC in imaging studies during a craving experience [26].

More than 700 miRNAs have been detected in the rodent brain [27], yet, less than <1% of brain-derived miRNAs have been explored to determine their association with drug seeking. Moreover, investigation of miRNA expression in serum exosomes derived from peripheral blood represents an intriguing avenue for biomarkers associated with drug craving. However, the profile of miRNAs in discrete brain regions has not been compared to blood miRNAs levels following heroin exposure, nor have blood miRNA levels been correlated to opioid seeking behavior. Thus, identifying miRNAs and their associated downstream protein pathways that are regulated in the brain as a result of chronic opioid exposure represents a novel strategy for determining previously understudied mechanisms that may have therapeutic relevance for the reduction of opioid seeking in OUD. Brain-region specific and drug dose-dependent regulation of miRNAs occurs [7], which necessitates the need to uncover the miRNA profile that results following a wide range of drug exposure protocols. In the present study, we have begun addressing these critical issues by performing small RNA sequencing of miRNAs and protein profiling on the OFC of rats that self-administered heroin at a dose of 0.03 mg/kg/infusion and underwent forced abstinence for 2 days (early abstinence). We chose to study miRNAs associated with heroin seeking behavior due to the association of miRNA expression with heroin dependence in human subjects [28–32]. Our results have uncovered a unique profile of drug-specific and sucrose-specific OFC miRNA and protein regulation in the acute abstinence period. We report select blood miRNA patterns may be robustly responsive to heroin self-administration and provide insight into drug-induced miRNA expression that has utility for biomarker measurement of heroin-taking behavior.

## Methods

### Subjects

35 adult male Sprague Dawley rats (Charles River) were used in this study. Rats were ∼240 g and 8 weeks old on arrival. All animals were pair-housed on a reverse light/dark cycle and provided food *ad libitum*, except where noted. Animals were acclimated to the facility for at least 5 days prior to beginning behavioral testing. All procedures were approved by Temple University’s Institutional Animal Care and Use Committee and followed the National Institute of Health’s Guide for the Care and Use of Laboratory Animals.

### Reagents

Heroin hydrochloride was obtained from the NIDA Drug Supply Program and dissolved in 0.9% sterile saline. 45 mg chocolate-flavored sucrose pellets were obtained from Bio-Serv (Flemington, NJ, USA).

### Self-administration

Self-administration of 0.03 mg/kg/infusion heroin and sucrose on an FR1 schedule was performed as previously reported [6, 24, 33]. Self-administration data for heroin animals was previously reported [24]. Drug-naïve animals were handled daily but did not undergo self-administration of any substance. 48-hours after the last heroin or sucrose session, animals were euthanized with 5% isoflurane and rapidly decapitated. Brains were immediately frozen in ice-cold liquid isopentane on dry ice and stored at −80°C until dissection.

### Blood & serum collection

Trunk blood was collected immediately following decapitation into 50 mL tubes and stored +4°C for ∼12 h. Following coagulation, blood was centrifuged at 2,000 rpm for 10 min. The serum supernatant was collected and stored at −80°C until RNA extraction.

### RNA extraction

Bilateral tissue punches of the OFC were collected from each animal. The regions of the OFC dissected were the ventral OFC and lateral OFC subregions. For extraction of total RNA from OFC tissue, the miRVANA PARIS RNA extraction kit (Life Technologies, Carlsbad, CA) was used, as we have previously reported [7, 24, 34]. Exosomal RNA was extracted from blood serum using the SeraMir Exosome RNA Amplification Kit (System Biosciences, Palo Alto, CA), according to the manufacturer’s instructions.

### Small-RNA sequencing

Library preparation and small-RNA sequencing of miRNAs was performed on individual biological replicate samples, 4 per group, by BGI Genomics (BGI Americas Corp, Cambridge, MA, United States), as we have previously described [24]. Briefly, RNA integrity >7.5 and 28S/18S>1.3 for each sample was confirmed with Bioanalyzer prior to library preparation. Small RNAs were size selected by PAGE gel, ligated with 3′ and 5′ adaptors and reverse transcribed to cDNA for PCR amplification with high-ping polymerase. Following PAGE gel separation, PCR products were purified and quantified by single strand DNA cyclization then DNA nanoballs were by rolling circle replication. DNA nanoballs were sequenced on the BGISEQ-500 and raw sequencing reads were filtered to yield clean reads without contamination. Clean reads were aligned to the reference genome with Bowtie2 [35]. The small-RNA seq yielded approximately 40 million reads per sample. Small RNA expression was calculated as transcripts per kilobase million (TPM). The open-access software miRPATH from DIANA was used to predict putative pathways of target genes impacted by heroin- or sucrose-associated miRNAs [36]. Raw sequencing data are available in the SRA and Gene Expression Omnibus repositories (Accession # PRJNA949640 and GSE237409). A list of OFC miRNA statistics between heroin, sucrose and naïve animals can be found in Supplementary Tables SE1–SE3.

### Proteomics

For proteomic profiling of OFC proteins following heroin or sucrose self-administration, dissected OFC tissue punches were obtained from 2 to 3 individual animals per group and submitted to the Core Research Facility at Yale University. Samples were processed and differential proteins were calculated, as we have previously reported [24]. Briefly, chloroform-methanol precipitation, dual enzymatic digestion with lysine and trypsin, acidification with 20% trifluoroacetic acid and desalting were performed on protein tissue samples prior to Label-Free Quantification with an Orbitrap Fusion Mass Spectrophotometer (ThermoFisher Scientific). Only proteins that were present in all samples were considered for comparison between two groups. KEGG pathway analysis of differentially regulated proteins between two comparisons were performed using DAVID (NIH) [37, 38]. Lists of protein expression values for and statistics of differentially expressed proteins between heroin, sucrose or drug-naïve animals is available in Supplementary Tables SE4–SE7. For overlap of miRNA data with proteomics, the microTCDS software from DIANA was used to identify putative targets of the heroin-regulated miRNAs [39].

### qPCR analysis

For measurement of serum miRNAs with qPCR, 20 ng of RNA was reverse transcribed into cDNA using the miRCURY LNA RT KIT (Qiagen), according to the manufacturer’s instructions, as we have previously reported [7, 24]. cDNA was diluted 1:60 and used as a template for qPCR with the miRCURY LNA SYBR Green PCR Kit (Qiagen) and the following LNA miRCURY SYBR green assays (Qiagen): rno-miR-877-5p (Assay ID: YP00205626); rno-miR-376a-3p (Assay ID: YP00205059); rno-miR-29c-3p (Assay ID: YP00204729); rno-miR-379-5p (Assay ID: YP00205658); rno-miR-186-5p (Assay ID: YP00206053); rno-miR-107-3p (Assay ID: YP00204468); rno-miR-219a-5p (Assay ID: YP00204780); rno-miR-451-5p (Assay ID: YP02119305); rno-miR-135a-5p (Assay ID: YP00204762); rno-miR-218b (Assay ID: YP02101069). rno-miR-320-3p (Assay ID: YP00206042) and rno-mir-191a-5p (Assay ID: YP00204306) were used as endogenous control genes because they were not regulated in the small-RNA sequencing analysis. Expression levels were calculated using 2^−ΔΔCT^ method [40].

### Statistical analysis

Two-tailed Mann-Whitney tests were used to confirm a significant preference of the reward-paired active lever during self-administration, compared to the inactive lever. For small-RNA sequencing, DEseq2 [41] was used to determine miRNAs differentially expressed between two groups, with the Benjamini and Hochberg method applied to correct for multiple comparisons [42]. miRNAs were considered statistically significant if the adjusted *p*-value between two groups was <0.05. For proteomics and miRNA qPCR, unpaired t-tests were used to determine differentially expressed proteins or miRNAs between treatment groups with normal distribution, with *p* < 0.05 considered significant. Two-tailed Mann-Whitney tests were used to compare miRNA expression when data was not normally distributed. Shapiro-Wilk normality tests were used to determine the distribution of data. Outliers were defined as values exceeding the mean by >2.5 times the standard deviation. Pearson correlations were used to compare the relationship between miRNA expression and drug-seeking behavior. All statistical analyses were performed using Graphpad Prism (Prism version 9, San Diego, CA, USA).

## Results

**FIGURE 1:**
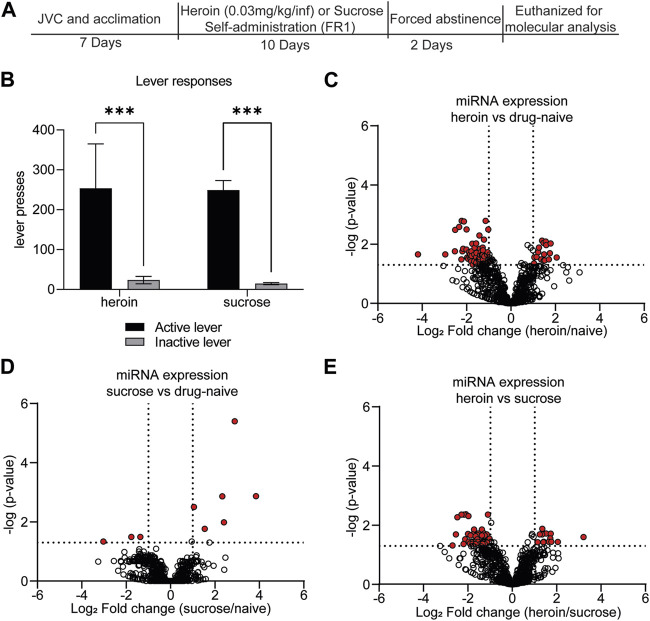
Self-administration of rewarding substances induces differential regulation of OFC miRNAs. **(A)** Experimental overview. Animals underwent acclimation and/or jugular vein catheterization (JVC). Seven days later, animals self-administered either heroin (0.03 mg/kg/infusion) for 6 h per day or sucrose pellets for 2 h per day for a total of 10 days. Animals underwent 2 days forced abstinence and were euthanized for molecular analysis of OFC and blood expression. **(B)** The average number of active or inactive lever responses across 10 days of self-administration of heroin or sucrose. *N* = 11–12/group. ***p < 0.001. Error bars depict ± the standard error of the mean (SEM). **(C–E)** Volcano plots depicting miRNA expression in the OFC for heroin vs. drug-naïve animals **(C)**, sucrose vs. drug-naïve animals **(D)**, or heroin vs. sucrose animals **(E)**. Red dots indicate miRNAs that were significantly regulated in each of the comparisons. Dotted lines indicate the threshold for significance based on *p*-value (horizontal) or log_2_ fold change (vertical).

**FIGURE 2:**
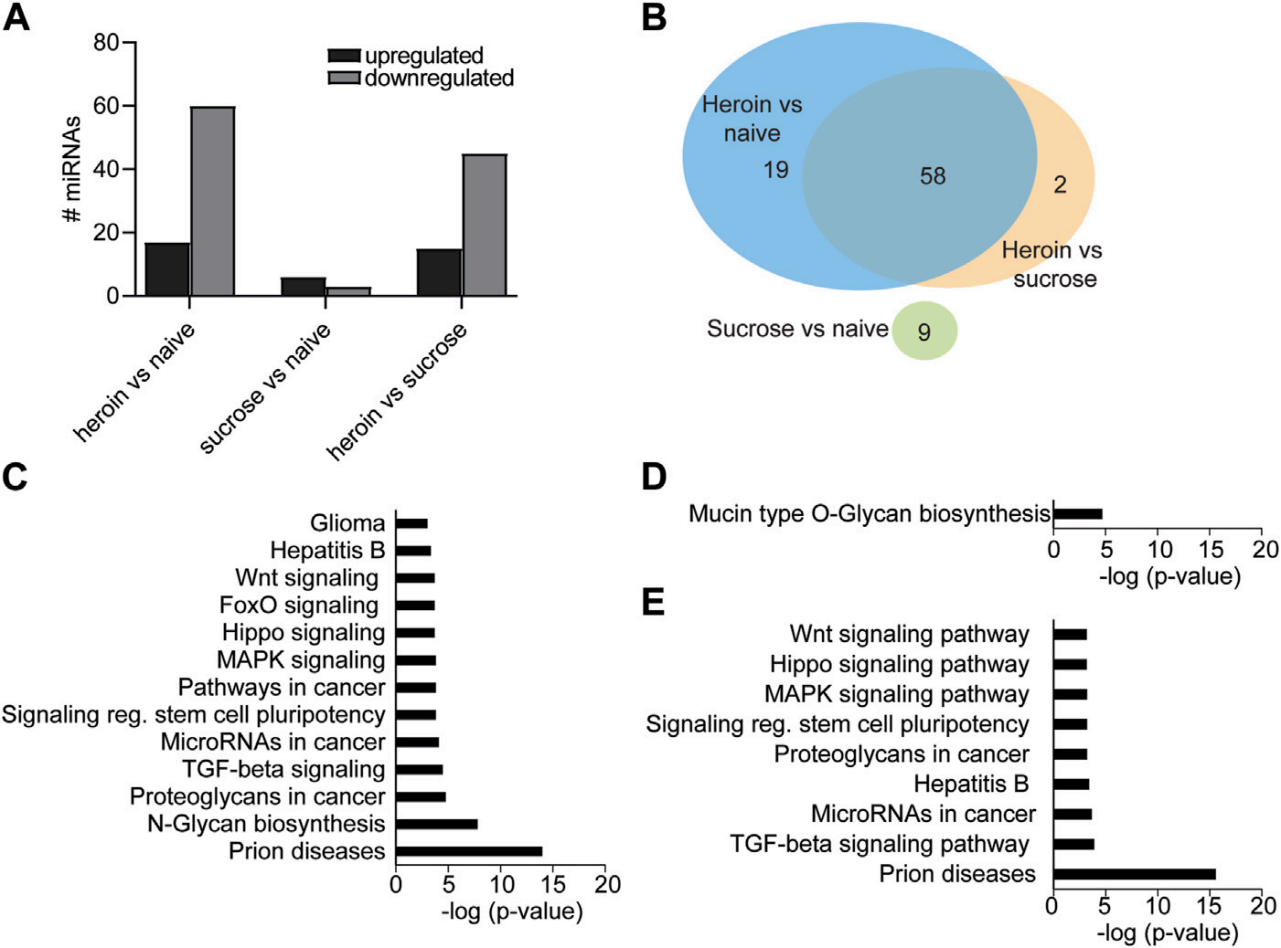
miRNA pathways in the OFC are regulated by acute withdrawal from heroin self-administration. **(A)** Number of miRNAs upregulated or downregulated following heroin or sucrose self-administration. **(B)** Venn diagram depicting the number of miRNAs that were overlapping or unique in the comparisons between heroin, sucrose, and naïve animals. **(C–E)** KEGG pathway terms of genes predicted to be targeted by miRNAs that were significantly enriched between heroin and naïve animals **(C)**, sucrose and naïve **(D)**, or heroin and sucrose **(E)**.

Our lab and others have demonstrated that self-administration of 0.03 mg/kg/infusion of heroin results in perseverant heroin-seeking behavior [6, 43], defined as a significant preference for the active, drug-paired lever compared to the inactive lever during cue-induced relapse sessions. Furthermore, such a protocol induces biochemical changes in the brain that recapitulate heroin-induced neuroadaptations observed in human subjects [43–46]. We sought to further characterize the neurobiological consequences of heroin self-administration at this dose by profiling miRNAs and proteins in the OFC, a brain region critical for opioid-seeking behavior [24, 25, 47, 48]. Results were compared to a separate group of rats that only self-administered sucrose pellets. Adult male Sprague Dawley rats self-administered heroin or sucrose pellets on an FR1 schedule for 10 days (Figure 1A). Rats in both heroin and sucrose groups demonstrated a significant preference for the active reward-paired lever compared to an inactive lever (Mann-Whitney test, heroin: U = 6, *p* < 0.0001; sucrose: U = 0, *p* < 0.0001; Figure 1B). Two days after the last self-administration session, animals were euthanized for small-RNA sequencing of OFC miRNAs or proteomic analysis of OFC proteins. Self-administration of heroin or sucrose resulted in differential expression of miRNAs in the OFC (Figures 1C–E; Supplementary Tables S1–S3). Exposure to heroin regulated 77 OFC miRNAs compared to drug-naïve animals, while sucrose only regulated 9 (Figures 2A, B). None of the sucrose-associated miRNAs were commonly regulated by heroin. The top 5 most regulated miRNAs in each comparison can be found in Table 1. Most heroin-regulated miRNAs were downregulated, suggesting a relief of miRNA inhibition of protein expression (Figure 2A). ∼75% of the OFC miRNAs regulated between heroin and naïve animals were also regulated between heroin and sucrose animals, demonstrating that heroin induces a unique profile of OFC expression beyond that observed with an appetitive reward (Figure 2B). Because miRNAs regulate mRNA translation into protein, we performed bioinformatic analysis to determine the putative pathways that are regulated by heroin- or sucrose-associated miRNAs. Predicted targets of heroin-associated miRNAs are involved in signaling pathways related to “Prion diseases,” “N-Glycan biosynthesis,” “Proteoglycans in cancer,” and “TGF-beta signaling,” among others (Figure 2C). Only one pathway was enriched for predicted target genes of the 9 sucrose-associated miRNAs: “Mucin type O-Glycan biosynthesis” (Figure 2D). Pathways predicted to be targeted by miRNAs significantly regulated between heroin and sucrose animals were largely overlapping with heroin-associated miRNAs and included the most significant pathway, “Prion diseases” (Figure 2E). Genes predicted to be targeted by miRNAs in this pathway included many with known links to opioid exposure, including *Elk1*, *Egr1*, and *Erk1* (Table 2) [44, 49–54].

**TABLE 1:** Top miRNAs regulated by heroin and sucrose.

Top 5 miRNAs regulated by heroin relative to naïve animals			
miRNA	miRBase Accession	*p*-value, adj	Log2FC
rno-miR-10b-5p	MIMAT0000783	0.022	−4.192
rno-miR-19a-3p	MIMAT0000789	0.022	−2.964
rno-miR-764-3p	MIMAT0017370	0.017	−2.527
rno-miR-29c-3p	MIMAT0000803	0.003	−2.516
rno-miR-377-3p	MIMAT0003123	0.003	−2.338

Top 5 miRNAs regulated by sucrose relative to naïve animals			
miRNA	miRBase Accession	*p*-value, adj	Log2FC
rno-let-7a-5p	MIMAT0000774	0.046	−3.031
rno-miR-214-3p	MIMAT0000885	0.001	2.331
rno-miR-196a-5p	MIMAT0000871	0.010	2.403
rno-miR-133a-3p	MIMAT0000839	<0.001	2.888
rno-miR-196b-5p	MIMAT0001082	0.001	3.849

Top 5 miRNAs regulated by heroin relative to sucrose animals			
miRNA	miRBase Accession	*p*-value, adj	Log2FC
rno-miR-19a-3p	MIMAT0000789	0.048	−2.707
rno-miR-764-3p	MIMAT0017370	0.020	−2.557
rno-miR-29c-3p	MIMAT0000803	0.005	−2.490
rno-miR-377-3p	MIMAT0003123	0.004	−2.302
rno-miR-183-5p	MIMAT0000860	0.025	3.193

**TABLE 2:** miRNA-mediated protein pathways enriched for ‘Prion Disease’ in heroin animals, relative to both naïve and sucrose comparisons.

Putative miRNA-targeted genes in “Prion Disease” pathway, commonly regulated by heroin vs. naïve or sucrose		
Ensembl ID	Gene name	miRNA
ENSRNOG00000007697	*C8a*	rno-miR-764-3p
ENSRNOG00000019422	*Egr1*	rno-miR-300-3p
ENSRNOG00000010171	*Elk1*	rno-miR-495, rno-miR-873-5p
ENSRNOG00000000596	*Fyn*	rno-miR-495
ENSRNOG00000018294	*Hspa5*	rno-miR-495, rno-miR-379-5p
ENSRNOG00000004575	*Il1a*	rno-miR-495, rno-miR-30e-5p, rno-miR-543-3p, rno-miR-758-3p
ENSRNOG00000002680	*Lamc1*	rno-miR-340-5p, rno-miR-764-3p, rno-miR-29a-3p, rno-miR-29b-3p, rno-miR-29c-3p
ENSRNOG00000019601	*Mapk3 (Erk1)*	rno-miR-15a-5p
ENSRNOG00000031890	*Ncam1*	rno-miR-466b-5p
ENSRNOG00000002126	*Ncam2*	rno-miR-340-5p, rno-miR-127-5p
ENSRNOG00000019322	*Notch1*	rno-miR-340-5p
ENSRNOG00000003696	*Prkx*	rno-miR-495, rno-miR-873-5p, rno-miR-3065-5p
ENSRNOG00000021259	*Prnp*	rno-miR-107-5p, rno-miR-466b-5p
ENSRNOG00000021164	*Stip1*	rno-miR-340-5p

**FIGURE 3:**
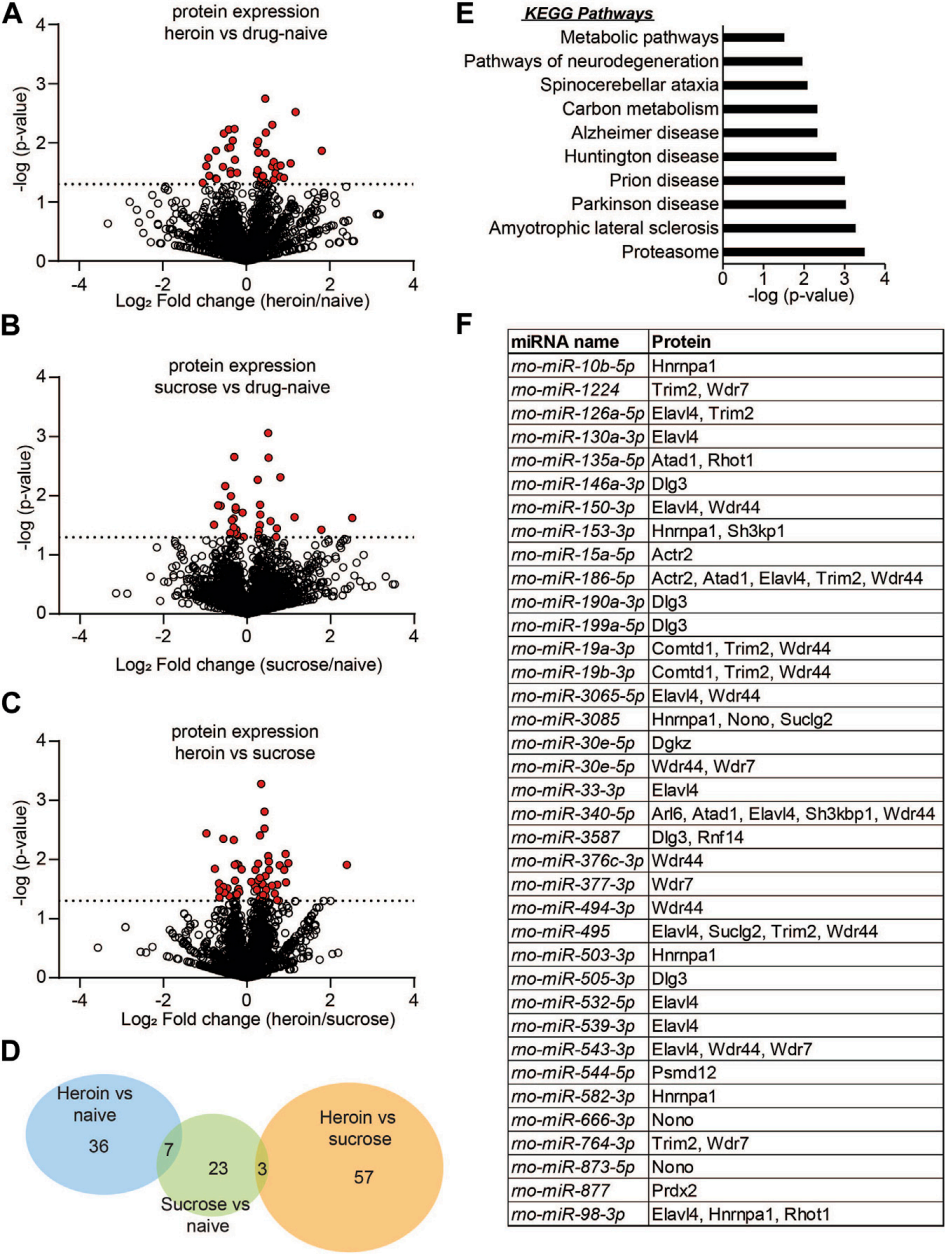
Protein expression is regulated in the OFC following acute abstinence from heroin self-administration. **(A–C)** Volcano plots depicting protein expression in the OFC for heroin vs. drug naïve animals **(A)**, sucrose vs. drug-naïve animals **(B)**, or heroin vs. sucrose animals **(C)**. Red dots indicate proteins that were significantly regulated in each of the comparisons. Dotted line indicates the threshold for significance based on *p*-value. **(D)** Venn diagram depicting the number of proteins that were regulated in each comparison of **(A–C)**. **(E)** KEGG pathway terms of proteins that were significantly enriched between heroin and naïve animals. **(F)** Overlap of miRNA sequencing data with proteomics to depict the significantly regulated miRNAs that are predicted to target significantly regulated proteins following chronic heroin.

To provide more insight into both the reproducibility of our initial miRNA sequencing findings as well as determine the potential miRNA-mediated protein pathways that are associated with heroin or sucrose self-administration, we performed parallel proteomics profiling on OFC tissue from separate animals that self-administered heroin or sucrose (Figure 3). More than 2,000 proteins were detected in the OFC with label-free mass spectrometry and heroin regulated expression of 43 OFC proteins relative to naïve animals and 60 OFC proteins relative to sucrose animals, while sucrose regulated expression of 33 OFC proteins relative to naïve animals (Figures 3A–D; Supplementary Tables S4–S7). 36 proteins were specifically regulated by heroin and not sucrose when compared to drug-naïve animals, while 57 proteins were specifically regulated by heroin and not sucrose when comparing to only sucrose animals (Figure 3D). 23 proteins were regulated by sucrose alone and not overlapping with heroin-exposed animals (Figure 3D). ∼60% of heroin-associated proteins were upregulated (Figures 3A, C), in contrast to the large downregulation of OFC miRNAs, suggesting that heroin may repress expression of some miRNAs to allow for positive gene expression regulation. The top 5 proteins regulated in each comparison can be found in Table 3. None of the proteins regulated in the comparison of heroin to naïve animals were overlapping in the comparison of heroin to sucrose animals. However, 7 proteins were commonly regulated by both heroin and sucrose when each was compared to drug-free naïve animals (Figure 3D; Table 4). The pathways of the heroin-regulated proteins included “Proteosome,” and several neurodegenerative pathways that all contained the similar proteins, such as “Amyotrophic lateral sclerosis,” “Parkinson disease” and “Prion disease,” “Huntington disease” and “Alzheimer disease” (Figure 3E). The only pathway significantly enriched for sucrose-associated OFC proteins was “Metabolic pathways” (Supplementary Figure S1). Proteins regulated by heroin compared to sucrose were significantly enriched in terms such as “Amyotrophic lateral sclerosis,” “Mineral absorption,” “Tight junction” and “Huntington disease” (Supplementary Figure S1). We overlapped the heroin-regulated OFC proteomics dataset with the small-RNA sequencing data of OFC miRNAs regulated following 2D forced abstinence from heroin and observed a high degree of overlap between the datasets (Figure 3F). Nearly two-thirds of the heroin-induced proteins are predicted to be regulated by a miRNA-mRNA interaction and approximately half of the heroin-regulated miRNAs targeted at least one mRNA for a heroin-associated protein (Figure 3F). This data indicates that heroin regulates OFC miRNA pathways.

**TABLE 3:** Top proteins regulated by heroin or sucrose.

Top 5 proteins regulated by heroin relative to naïve animals			
Uniprot Accession	Protein Description	Protein Symbol	Log2FC
Q3ZAU6	RBR-type E3 ubiquitin transferase	Rnf14	−1.043
A0A0G2JSH9	Peroxiredoxin 2	Prdx2	−0.959
P40307	Proteasome subunit beta type-2	Psmb2	1.059
D3ZAF6	ATP syntdase subunit f, mitochondrial	Atp5mf	1.179
A0A0G2K707	Diacylglycerol kinase	Dgkz	1.806

Top 5 proteins regulated by sucrose relative to naïve animals			
Uniprot Accession	Protein Description	Protein Symbol	Log2FC
P32232	Cystatdionine beta-syntdase	Cbs	−0.794
P11348	Dihydropteridine reductase	Qdpr	0.795
Q62785	28 kDa heat- and acid-stable phosphoprotein	Pdap1	1.138
Q6QIX3	Probable proton-coupled zinc antiporter	Slc30a3	1.777
M0R4L7	Histone Cluster 1 H2B Family Member L	Hist1h2bl	2.518

Top 5 proteins regulated by heroin relative to sucrose animals			
Uniprot Accession	Protein Description	Protein Symbol	Log2FC
Q6QIX3	Probable proton-coupled zinc antiporter	Slc30a3	−0.970
F1MAH8	CAP-Gly domain-containing linker protein 1	Clip1	0.923
Q6MGC4	H2-K region expressed gene 2, rat ortdologue	Pfdn6	0.933
A0A0G2K4W2	Transcription factor BTF3	Btf3	0.990
Q6IRG7	Claudin	Cldn11	2.388

**TABLE 4:** Proteins commonly regulated by heroin or sucrose comparisons.

Commonly regulated by sucrose relative to drug-naïve or heroin animals			
Uniprot Accession	Protein Description	Protein Symbol	Length
D3ZAN3	Alpha glucosidase 2 alpha neutral subunit (Predicted)	Ganab	797 AA
Q920Q0	Paralemmin-1	Palm	383 AA
Q6QIX3	Probable proton-coupled zinc antiporter SLC30A3	Slc30a3	388 AA

Commonly regulated by heroin and sucrose relative to drug-naïve animals			
Uniprot Accession	Protein Description	Protein Symbol	Length
P09117	Fructose-bisphosphate aldolase C	Aldoc	363 AA
B1WC73	ADP-ribosylation factor-like protein 6	Arl6	186 AA
A0A0H2UHI7	ATPase family, AAA domain containing 1	Atad1	403 AA
D3ZM21	Catechol-O-metdyltransferase domain containing 1	Comtd1	262 AA
P47942	Dihydropyrimidinase-related protein 2	Dpysl2	572 AA
A0A140TAF2	ELAV-like protein 4	Elavl4	385 AA
Q925Q9	SH3 domain-containing kinase-binding protein 1	Sh3kbp1	709 AA

**FIGURE 4:**
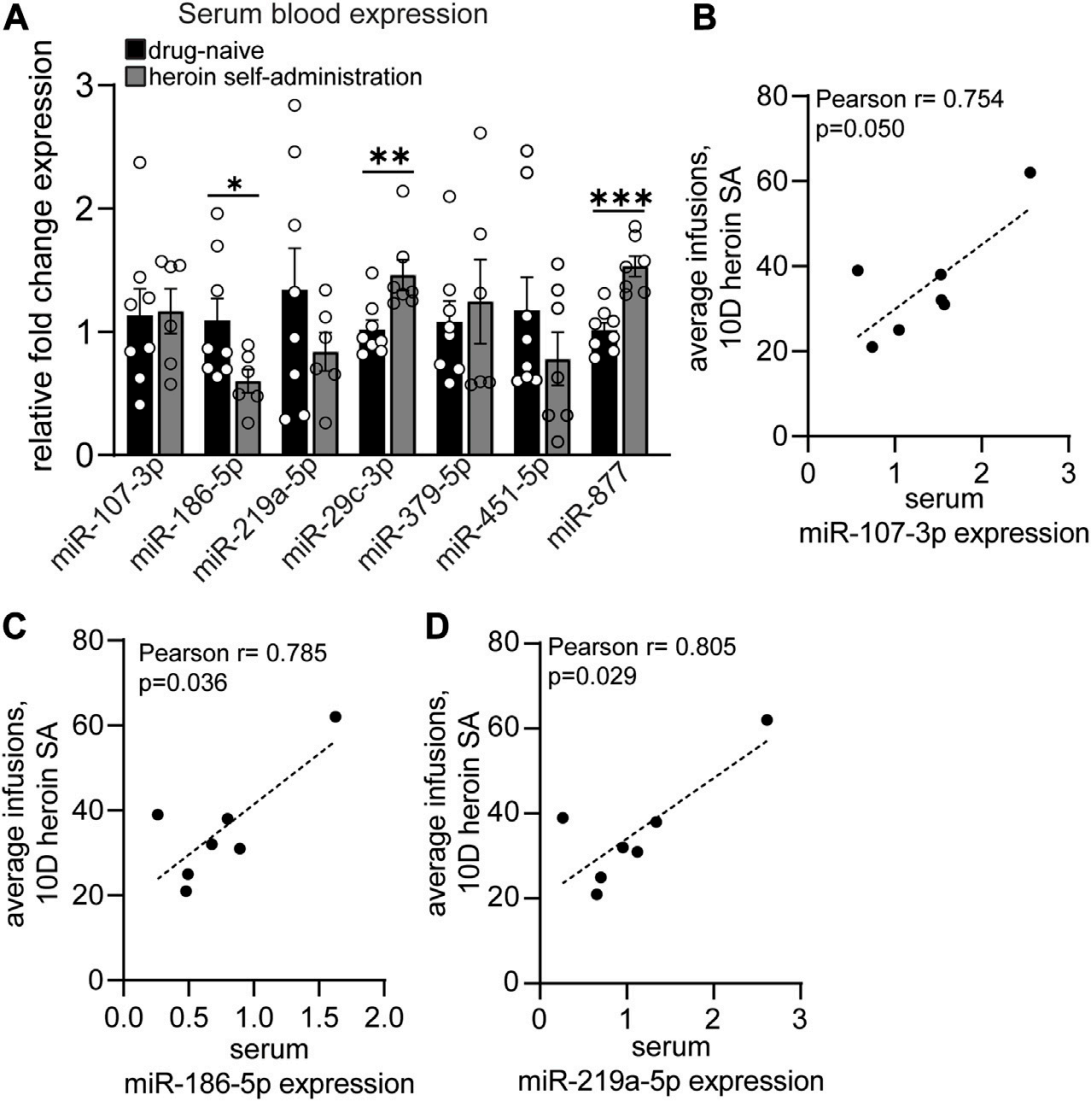
Acute withdrawal from heroin self-administration induces miRNA expression regulation in the blood that reflects the OFC profile. **(A–D)** Serum blood expression of heroin-associated miRNAs measured by qPCR in drug-naïve and heroin self-administration animals. **(B–D)** Correlations between serum miRNA expression and the average number of heroin infusions over the course of 10 days of self-administration. *N* = 6–8/group. *p < 0.05. **p < 0.01. ***p < 0.001. Error bars depict ± SEM.

To provide insight into the potential biomarker utility of heroin-associated OFC miRNAs, we examined the expression patterns of a subset of these miRNAs in peripheral blood samples. Using RNA extracted from exosomes in serum blood samples, we performed qPCR to measure expression of 10 miRNAs that were regulated in the OFC: miR-107-3p; miR-135a-5p; miR-186-5p; miR-218b; miR-219a-5p; miR-29c-3p; miR-376a-3p; miR-379-5p; miR-451-5p; miR-877-5p (Figure 4A). These miRNAs were chosen based on their robust expression values in the central nervous system as well as their high fold change values in the OFC. Three of the miRNAs, miR-135a-5p, miR-218b, and miR-376a-3p, had very low expression in serum samples and were not able to be quantified. Of the remaining 7 miRNAs we examined, miR-186-5p was significantly downregulated (t [12] = 2.179; *p* = 0.050) and both miR-29c-3p and miR-877-5p were significantly upregulated (miR-29c-3p: Mann-Whitney U = 5, *p* = 0.006; miR-877-5p: unpaired t-test: t [13] = 5.115; *p* = 0.0002) (Figure 4A). The regulation of miR-186-5p and miR-877 mirrored the opioid-induced differential regulation of these two miRNAs observed in the OFC. Expression levels of three miRNAs positively correlated with heroin infusions administered on the last day of self-administration: miR-107-3p (Pearson r = 0.754; *p* = 0.050), miR-186-5p (Pearson r = 0.785; *p* = 0.036) and miR-219a-5p (Pearson r = 0.805; *p* = 0.029) (Figures 4B–D). These data demonstrate that blood miRNA levels may, in some instances, reflect heroin-induced regulation of OFC miRNAs.

## Discussion

Opioid exposure results in brain-region specific regulation of the miRNA profile. Our results demonstrate that acute abstinence from heroin self-administration results in a robust regulation of both miRNAs and proteins in the OFC. In a prior study, we determined that heroin self-administration induces lasting regulation of OFC miRNAs that may be manipulated to modulate long-lasting heroin seeking behavior [24]. The present work identified a unique profile of OFC miRNA regulation during acute abstinence that greatly differed from that observed in the OFC following late abstinence. This data demonstrate that the brain undergoes neuroadaptations following cessation of drug use and the best miRNA pathways to target pharmacologically for reduction of drug seeking behavior may be dynamically regulated in a time-dependent manner, as we have previously observed for morphine exposure [7]. Of the 77 heroin-associated miRNAs that were identified as differentially regulated in the OFC between heroin and naïve animals, we determined that none of the miRNAs were overlapping with the OFC profile following between sucrose self-administration and naive. However, we identified 7 proteins commonly regulated in the OFC following heroin or sucrose self-administration relative to naïve animals. These data suggest that the profile of heroin-associated miRNAs we identified is likely due to drug exposure and not learning a rewarding task. In addition, the common proteins regulated by both sucrose and heroin may be due to miRNA-independent pathways, or the unique profile of miRNAs regulated by sucrose and heroin commonly target the same OFC proteins.

In comparison with our previously published study that examined miRNA regulation associated with long-lasting heroin seeking behavior following 21D forced abstinence from either 0.03 mg/kg/infusion or 0.075 mg/kg/infusion heroin, we observed some overlap of heroin-regulated miRNA expression in the OFC. 5 miRNAs were commonly regulated following 2 or 21D forced abstinence from the 0.03 mg/kg/infusion heroin dose: miR-219a-5p, miR-299a-5p, miR-29c-3p, miR-666-3p and miR-764-3p. In addition to miR-219a-5p, miR-218b, miR-3065-5p, miR-338-3p, miR-379-5p and miR-503-3p, which were regulated in the present study following 2D forced abstinence from 0.03 mg/kg/infusion heroin, were also regulated in the OFC following 21D forced abstinence from a higher heroin dose of 0.075 mg/kg/infusion. The combination of these two studies demonstrates that some miRNAs, such as miR-219a-5p, are regulated by both high and low doses of heroin. Moreover, a subset of miRNAs are regulated immediately following heroin and remain altered for at least 21D following the last heroin self-administration session. This later finding demonstrates that miRNA regulation in the OFC is a long-lasting neuroadaptation that results from chronic heroin exposure. The dynamic responsiveness of miRNAs to opioids is likely dependent on drug dose, timepoint and region specificity. However, the present study is limited in that it does not address the contribution of the aforementioned variables on heroin-induced miRNA expression. Future studies that include animals of both sexes, additional timepoints following drug exposure, profiling of multiple brain regions and variable periods of drug exposure that may more accurately model physical dependence are likely to yield additional insight into the impact of heroin on miRNA expression. Validation of RNA-sequencing and proteomics with secondary measures will also help to narrow down the most relevant miRNAs for support of drug seeking behavior. While our study did not perform secondary validation with qPCR or western blots, the sequencing and proteomics datasets were obtained from separate groups of animals, yet we still observed overlap of putative heroin-regulated miRNA pathways with the proteomics data (Figure 3F).

The correspondence of the “Prion disease” pathway enriched for proteins regulated by heroin, as well as putative gene targets of miRNAs regulated by heroin, demonstrates the reproducibility of our findings. The genes predicted to be regulated in the “Prion disease” pathway by heroin-associated miRNAs included several transcription factors that have been demonstrated to regulate expression of proteins observed in our heroin-associated protein list, including Atp5pd (*Elk1*) and Uqcrfs1 (*Elk1*) [55]. These results are not surprising, given that the KEGG entry for the “Prion disease” pathway includes many genes known to be involved in drug-induced neuropathologies, including Erk1/2, CREB, Egr1, p38/JNK, GSK-3B, PKA, Fyn, and other genes involved in proteosomal and mitochondrial function [43, 44, 49–51, 56–59]. However, by describing a pattern of genes regulated by heroin-associated miRNAs in the OFC, our study begins to fill in the molecular gap between heroin exposure and heroin-induced neuroadaptations. These findings suggest that miRNAs may function as key modulators of heroin-regulated proteins.

Published studies have reported differential regulation of miRNA expression in peripheral blood samples from humans exposed to opioids [28, 30, 60–64]. Demonstration of the utility of detecting miRNA expression in peripheral blood samples is evidenced by the observation that miRNAs may be predictive of need for hospitalization or pharmacological interventions in opioid-exposed infants [62]. However, it is unclear how the blood miRNA profile reflects brain-region specific miRNA expression induced by drugs. Only one such study has been performed with human samples, and this is likely due to the challenges of collecting blood and postmortem samples in a timely manner. Grimm et al reported the correspondence of frontal cortex brain and blood miRNA levels in postmortem human samples from OUD subjects and observed a large overlap in miRNA expression [65]. Of the miRNA profile measured in the BA9 region, the authors observed differential expression of hsa-miR-10b-5p, hsa-miR-337-3p, has-miR-340-5, hsa-miR-376a-3p, hsa-miR-376b-3p, hsa-miR-379-5p, hsa-miR-486-3p, hsa-miR-495-3p, and hsa-miR-758-3p [65], which were all dysregulated in the OFC of heroin-exposed rats in the current study. Investigation into the relationship between drug exposure and regulation of brain miRNAs that can also be detected in the periphery can be accomplished easily with rodent models of self-administration but has yet to be done. We report for the first time the regulation of two miRNAs, miR-186-5p and miR-877, in both the OFC and the serum of animals that have previously self-administered heroin. We identified three miRNAs that correlated with heroin infusions-miR-186-5p, miR-107-3p and miR-219a-5p-which suggests that miRNAs may have putative biomarker utility for understanding drug motivation or abstinence behavior. Indeed, miR-186-5p was significantly reduced in both the OFC and serum of heroin-exposed animals in our study, as well in peripheral blood samples obtained from humans that meet criteria for OUD [63]. We also identified miR-451-5p as a miRNA downregulated in the OFC following heroin self-administration and this miRNA was significantly downregulated in blood exosomal samples from human patients with heroin use disorder [64]. Additional studies to understand the responsiveness of blood miRNA expression as an indication of opioid craving or recovery from OUD may help to inform patient care in the clinic.

## Data Availability

The datasets presented in this study can be found in online repositories. The names of the repository/repositories and accession number(s) can be found in the article/Supplementary Material.
