# Supplementary material for: MicroRNA-mediated translational pathways are regulated in the orbitofrontal cortex and peripheral blood samples during acute abstinence from heroin self-administration
Source: Adv Drug Alcohol Res. 2023 Aug 14;3:11668. doi: 10.3389/adar.2023.11668 (PMC10880771; doi:10.3389/adar.2023.11668)
Supplement: Supplementary file 1 [file DataSheet2.pdf]

Supplemental info:

Supplemental Figure 1: Identification of pathways significantly regulated following heroin or sucrose.

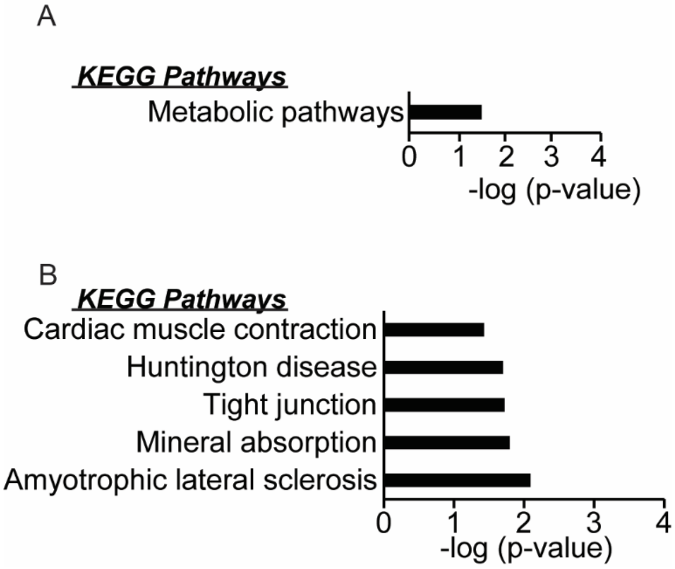

KEGG pathway terms of proteins that were significantly enriched between sucrose and naïve (A), or heroin and sucrose (B).

Table SE 1: list of OFC miRNA statistics between animals that self-administered heroin (0.03 mg/kg/infusion) for 10 days and drug-free naïve animals

Table SE 2: list of OFC miRNA statistics between animals that self-administered sucrose for 10 days and drug-free naïve animals

Table SE 3: list of OFC miRNA statistics between animals that self-administered heroin (0.03 mg/kg/infusion) or sucrose for 10 days

Table SE4: Average normalized intensity of proteins detected during proteomics for all sample groups.

Table SE 5: list of OFC protein expression statistics between animals that self-administered heroin (0.03 mg/kg/infusion) for 10 days and drug-free naïve animals

Table SE 6: list of OFC protein expression statistics between animals that self-administered sucrose for 10 days and drug-free naïve animals

Table SE 7: list of OFC protein expression statistics between animals that self-administered heroin (0.03 mg/kg/infusion) or sucrose for 10 days

Compressed Zip File: microRNA expression counts for each individual sample, obtained with RNA sequencing.
